# Task-irrelevant odours affect both response inhibition and response readiness in fast-paced Go/No-Go task: the case of valence

**DOI:** 10.1038/s41598-019-55977-z

**Published:** 2019-12-18

**Authors:** Javier Albayay, Umberto Castiello, Valentina Parma

**Affiliations:** 1 Department of General Psychology, University of Padova, Via Venezia 8, 35131 Padova, Italy; 2 Department of Psychology, Temple University, 1701 N 13th St, 19122 Philadelphia, PA United States of America; 3 Neuroscience Area, International School for Advanced Studies, Via Bonomea 265, 34151 Trieste, Italy

**Keywords:** Motor control, Human behaviour

## Abstract

Whether emotional stimuli influence both response readiness and inhibition is highly controversial. Visual emotional stimuli appear to interfere with both under certain conditions (e.g., task relevance). Whether the effect is generalisable to salient yet task-irrelevant stimuli, such as odours, remains elusive. We tested the effect of orthonasally-presented pleasant (orange) and unpleasant odours (trimethyloxazole and hexenol) and clean air as a control on response inhibition. In emotional Go/No-Go paradigms, we manipulated the intertrial interval and ratios of Go/No-Go trials to account for motor (Experiment 1, *N* = 31) and cognitive (Experiment 2, *N* = 29) response inhibition processes. In Experiment 1, participants had greater difficulty in withholding and produced more accurate and faster Go responses under the pleasant vs. the control condition. Faster Go responses were also evident in the unpleasant vs. the control condition. In Experiment 2, neither pleasant nor unpleasant odours modulated action withholding, but both elicited more accurate and faster Go responses as compared to the control condition. Pleasant odours significantly impair action withholding (as compared to the control condition), indicating that more inhibitory resources are required to elicit successful inhibition in the presence of positive emotional information. This modulation was revealed for the motor aspect of response inhibition (fast-paced design with lower Go/No-Go trial ratio) rather than for attentional interference processes. Response readiness is critically impacted by the emotional nature of the odour (but not by its valence). Our findings highlight that the valence of task-irrelevant odour stimuli is a factor significantly influencing response inhibition.

## Introduction

Motor response inhibition, defined as the ability to suppress inappropriate actions, allows individuals to flexibly navigate the world, generating adaptive responses under neutral and emotional conditions^1^. The use of Go/No-Go paradigms represents a well-established way to measure response inhibition in the laboratory. The Go/No-Go paradigm^2^ involves a continuously presented series of stimuli composed of frequent “Go” cues, to which subjects ought to respond with an action as rapidly as possible, and infrequent “No-Go” cues, to which subjects ought not to respond. The disparity in frequency between Go/No-Go cues creates a prepotent tendency to respond to the upcoming stimulus. When a No-Go cue appears, such prepotent response tendency must be withheld. The ability to inhibit an impending motor response is classically quantified as the rate of errors elicited in No-Go trials (failed-to-No-Go^3^). Other indices have been used to characterise aspects of motor performance in the Go/No-Go task. For instance, failing to produce an action when a Go cue is presented (failed-to-Go) is used to quantify attentional failures^4,5^ and the reaction times (RTs) for Go actions have been assumed to reflect different aspects of behavioural execution, such as (motor) response readiness^6^ and approach tendencies^4^.

### Go/No-Go task features affecting motor readiness and motor inhibition

The accurate performance to the Go/No-Go task requires the integration of motor and cognitive skills. This becomes evident when the cognitive resources necessary to accurately perform the task are taxed, as revealed by the experimental manipulation of intertrial intervals (ITI) and Go/No-Go ratios. Indeed, fast-paced Go/No-Go paradigms (ITI < 4 s^7^) challenge action withholding more than slow-paced designs^8^ while maintaining attentional demands stable on the Go cues^9^. Also, the Go/No-Go ratios affect the number of failed-to-No-Go responses, with lower proportions of No-Go cues producing higher number of failed-to-No-Go responses^7,8^. Altogether, fast-paced designs with infrequent No-Go trials elicit more prepotent motor tendencies on No-Go trials^7^ better accounting for motor response inhibition rather than other aspects of cognitive control (e.g., attentional interference and decision-making processes^9^).

### Effects of emotional stimuli on response readiness and response inhibition

Incorporating emotional contextual stimuli into Go/No-Go paradigms provides insights on how one’s emotional experience modulates action inhibition^5,10–12^ and accounts for the variety of inhibitory control mechanisms^13^. Emotional Go/No-Go paradigms preserve the basic neuropsychological constructs of the traditional (non-emotional) task, representing a valid measure of response inhibition^5^. Following the circumplex model of emotion^14^, the great part of emotional experiences can be categorised on the dimensions of arousal and valence. Emotion-induced arousal alters the allocation of attentional resources^15^ and heightens sensitivity to sensory cues necessary for accurate task performance^16^. In the context of motor response inhibition, the more arousing the stimuli presented (e.g., images), the greater the interference with action performance, as reflected by the increasing stopping latencies in function of the arousal of the images at a stop signal task (i.e., a paradigm used to measure the inhibition of already initiated responses^17^).

The role of valence in altering response inhibition is still not fully clarified. A series of possible explanations for the mixed results can be advanced. First, the findings from fast-paced Go/No-Go designs (those presenting greater cognitive load) reveal a valence effect on response inhibition (e.g.,^3,10^), whereas findings from slow-paced designs do not (e.g.,^18,19^). Second, the relevance of the affective cues for the task is sporadically influenced by valence. Mirabella^6^ found that fearful (vs. happy) faces increase both RTs (response readiness) and rates of failed-to-Go responses (attentional failures) exclusively when the emotional stimulus is task-relevant. However, task-irrelevant emotional stimuli have also been shown to modulate Go/No-Go performance, with faster RTs^3,10^ and more failed-to-No-Go responses^3^ following pleasant (vs. unpleasant) cues. All in all, it seems that the influence of emotional stimuli depends on a series of task requirements, calling for additional research on the influence of emotional stimuli on response inhibition.

### Decoupling response inhibition from interference control by using affective olfactory cues

The majority of the literature on emotion-modulated response inhibition uses brief presentations of visual stimuli, such as affective words^20–22^ and emotional facial expressions^6,17,23–25^, and sporadically auditory stimuli^19,26^ and flavours^3^. However, in ecological situations (e.g., cravings), behavioural control happens to be based on a variety of sensory information and mostly implicitly. Assuming that all sensory information at different levels of awareness similarly affects response inhibition would be misguided.

Olfaction is often labelled as a sensory modality that is naturally more emotional than vision. This idea is supported by the anatomical architecture of the olfactory system, which has within its first synapses the intersection with the limbic system (instead the distance of the limbic system and the visual system is much greater^27^). In line with this notion, Adolph and Pause^28^ recently demonstrated that odours elicited stronger emotional responses than comparable visual stimuli and proposed that perceptually triggered emotional responses are modality-dependent. This also seems to be the case for single odour exposures^29^.

Orthonasal olfactory cues offer an ideal test bed to evaluate how task-irrelevant emotional contexts affect emotional response inhibition^22^. Odours provide intense emotional information in the span of a sniff^30^, they are less subjected to labelling issues (i.e., verbalising an odour is normally a challenging task^31^), and they are more perceptually complex than most verbal and visual material^32^. Critically, they modulate motor control in accordance to their intrinsic properties (e.g., size^33^) and affective features (e.g.,^34,35^) and their valence influences decision making^36^. However, results in the context of motor readiness and motor inhibition are nonexistent or highly flawed^37–40^. To examine the influence of task-irrelevant orthonasal odours on response readiness and response inhibition, we presented a pleasant and an unpleasant odour (plus clean air as a control) to healthy controls performing a Go/No-Go task. In Experiment 1 the task was fast-paced (aimed at accounting for motor response inhibition) whereas in Experiment 2 the task was slow-paced (aimed at accounting for attentional interference processes).

### The present study

**Figure 1:**
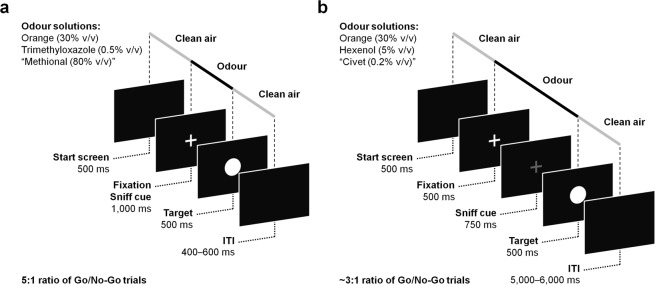
Graphical description of the emotional olfactory Go/No-Go task in (**a**) Experiment 1 and (**b**) Experiment 2 [the red + (sniff cue) is represented in grey colour]. In control trials only, clean air is presented continuously. The methional (in Experiment 1) and civet (in Experiment 2) odours were excluded from the main analyses (please refer to the Materials and Methods section for details about the odour stimuli).

Under the assumptions of the so-called spillover theory, based on which contexts with positive valence require a greater mobilisation of inhibitory resources to withhold responses^3^, in Experiment 1 we tested 31 participants and hypothesised that they would present greater difficulties in withholding a prepared motor response and a facilitation in response readiness (faster RTs and more accurate Go responses) under the exposure to a pleasant odour as compared to the other conditions (Fig. 1, panel a).

In Experiment 2, we investigated whether valence affects the interference control processes related to response inhibition by means of a slow-paced Go/No-Go task (i.e., giving more access to contextual information^9^ as compared to Experiment 1) in a sample of 29 participants. Overall, we expected low rates of failed-to-No-Go responses across odour conditions (i.e., floor effect; e.g.,^19^). On the other hand, we anticipate odours to alert the participants to the task and produce faster and more accurate Go responses as compared to the less salient control condition. Given the reduced cognitive load (longer ITI and higher proportion of No-Go cues), we did not expect the pleasant (vs. control and unpleasant) odour condition to facilitate response readiness (Fig. 1, panel b).

## Results

### Experiment 1 – fast-paced Go/No-Go task

#### Odour conditions are isointense and differ in pleasantness

**Figure 2:**
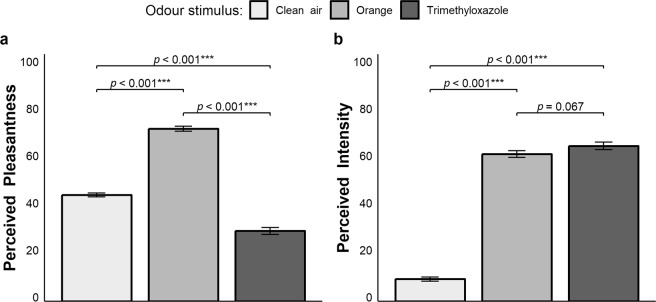
Results of Experiment 1. (**a**) Perceived pleasantness of the odour stimuli. (**b**) Perceived intensity of the odour stimuli.

The analysis on the subjective rating of pleasantness for the three odours conditions presented (i.e., clean air, orange and trimethyloxazole) showed a significant main effect of odour (Fig. 2, panel a), χ^2^(2) = 604.81, *p* < 0.001, AIC_RL_ > 100 (AIC = Akaike information criterion; please refer to the Statistical analyses section for more details), whereas the effect of cycle (i.e., times at which the participants rated the odour conditions), χ^2^(8) = 1.610, *p* = 0.991, AIC_RL_ < 0.001, and the interaction odour × cycle, χ^2^(16) = 8.454, *p = *0.934, AIC_RL_ < 0.001, were not significant. Multiple comparisons revealed that the trimethyloxazole odour (29.9 ± 24.5) was significantly more unpleasant than both orange (73.3 ± 18.1, *p* < 0.001) and clean air (45.2 ± 13.9, *p* < 0.001), whereas orange was significantly more pleasant than clean air (*p* < 0.001).

Regarding the perceived intensity, a significant main effect of odour was found (Fig. 2, panel b), χ^2^(2) = 933.21, *p* < 0.001, AIC_RL_ > 100, while neither the effect of cycle, χ^2^(8) = 12.186, *p* = 0.143, AIC_RL_ = 0.148, nor the interaction odour × cycle, χ^2^(16) = 8.191, *p* = 0.943, AIC_RL_ < 0.001, reached significance. Multiple comparisons showed that orange (62.5 ± 24.2) and trimethyloxazole (66.0 ± 26.0) did not differ significantly (*p* = 0.067), whereas clean air (9.4 ± 14.6) was significantly less intense than both odours (*p*-values < 0.001). Ratings’ average response time was 1620 ± 1460 ms. The fact that the effect of cycle was not significant, revealed that both perceived pleasantness and intensity of the odours were consistent throughout the experiment.

#### More accurate withholding when exposed to clean air as compared to a pleasant (but not unpleasant) odour

**Figure 3:**
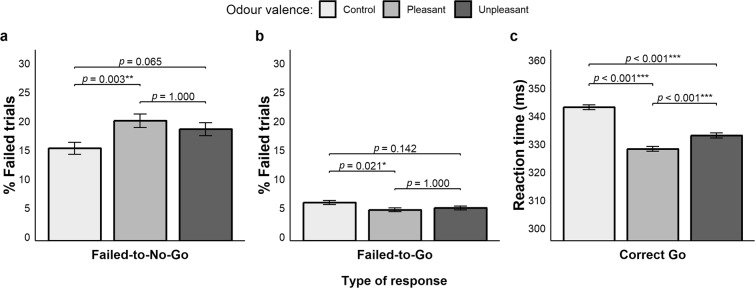
Results of Experiment 1. (**a**) Rates of failed-to-No-Go responses. (**b**) Rates of failed-to-Go responses. (**c**) Reaction times of correct Go responses. Error bars represent standard error of the mean.

A significant main effect of valence on failed-to-No-Go responses emerged (Fig. 3, panel a), χ^2^(2) = 11.065, *p* = 0.004, AIC_RL_ = 34.202. Multiple comparisons showed that participants were significantly less proficient in withholding inappropriate responses when a pleasant odour was delivered (21%, *p* = 0.005) as compared to the control condition (16%, *p* = 0.003); no other comparison reached significance: pleasant vs. unpleasant (19.3%, *p* = 1.000), although control vs. unpleasant (*p* = 0.065) showed a trend.

#### More accurate Go responses when exposed to a pleasant (but not unpleasant) odour as compared to clean air

The analysis of failed-to-Go responses showed a main effect of valence (Fig. 3, panel b), χ^2^(2) = 7.812, *p* = 0.020, AIC_RL_ = 6.726. Multiple comparisons revealed that participants were more accurate when primed with a pleasant odour (5.3%) as compared to the control conditions (6.6%, *p* = 0.021); other comparisons were not significant: control vs. unpleasant (5.7%, *p* = 0.142), pleasant vs. unpleasant (*p* = 1.000).

#### Faster Go responses when exposed to a pleasant odour as compared to an unpleasant odour and clean air

A significant main effect of valence on RTs of correct Go responses was retrieved (Fig. 3, panel c), χ^2^(2) = 191.33, *p* < 0.001, AIC_RL_ > 100. Multiple comparisons showed slower responses in the control condition (345 ± 60 ms) as compared to the pleasant (330 ± 58 ms, *p* < 0.001) and unpleasant ones (335 ± 59, *p* < 0.001). Participants were faster in the pleasant condition as compared to the unpleasant one (*p* < 0.001).

### Experiment 2 – slow-paced Go/No-Go task

#### Odour conditions are isointense and differ in pleasantness

**Figure 4:**
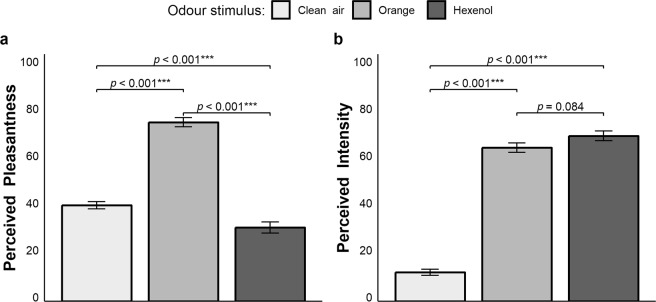
Results of Experiment 2. (**a**) Perceived pleasantness of the odour stimuli. (**b**) Perceived intensity of the odour stimuli.

As expected, the analyses revealed a significant main effect of odour on pleasantness (Fig. 4, panel a), χ^2^(2) = 251.77, *p* < 0.001, AIC_RL_ > 100, while neither the effect of cycle, χ^2^(4) = 1.142, *p* = 0.888, AIC_RL_ = 0.032, nor the interaction odour × cycle, χ^2^(8) = 2.869, *p = *0.942, AIC_RL_ = 0.001, were significant. Multiple comparisons showed that the hexenol odour (31.3 ± 28.3) was rated as more unpleasant as compared to orange (76.0 ± 23.4, *p* < 0.001) and clean air (40.8 ± 18.9, *p* < 0.001), whereas orange was perceived as more pleasant than clean air (*p* < 0.001).

As for intensity, a significant main effect of odour was retrieved (Fig. 4, panel b), χ^2^(2) = 448.14, *p* < 0.001, AIC_RL_ > 100, whereas the effect of cycle, χ^2^(4) = 4.445, *p* = 0.349, AIC_RL_ = 0.169, and the interaction odour × cycle, χ^2^(8) = 6.844, *p* = 0.553, AIC_RL_ = 0.010, did not reach statistical significance. Orange (65.3 ± 24.4) and hexenol (70.2 ± 24.9) were rated as isointense (*p* = 0.084), while clean air (12.3 ± 15.3) was perceived as less intense as compared to both odour conditions (*p*-values < 0.001). The average response time of the ratings was 1988 ± 837 ms.

#### Equally accurate withholding regardless of the olfactory context

**Figure 5:**
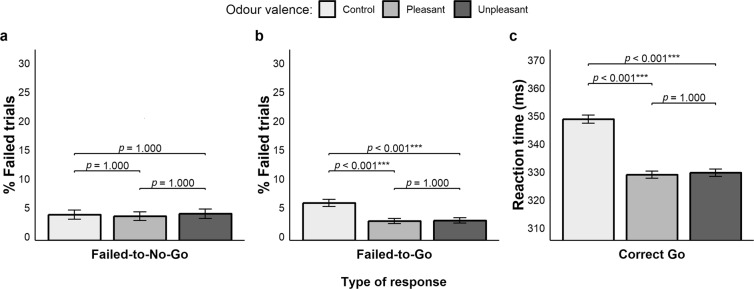
Results of Experiment 2. (**a**) Rates of failed-to-No-Go responses. (**b**) Rates of failed-to-Go responses. (**c**) Reaction times of correct Go responses. Error bars represent standard error of the mean.

The main effect of valence on the rate of failed-to-No-Go responses was not significant (Fig. 5, panel a), χ^2^(2) = 0.167, *p* = 0.920, AIC_RL_ = 0.147, suggesting that the olfactory manipulation did not have an impact on the interference control processes related to response inhibition (control = 4.4%; pleasant = 4.2%; unpleasant = 4.6%).

#### More accurate Go responses when exposed to an odour as compared to clean air regardless of valence

The main effect of valence on the rate of failed-to-Go responses was significant (Fig. 5, panel b), χ^2^(2) = 23.69, *p* < 0.001, AIC_RL_ > 100. Participants were more accurate when presented with pleasant (3.3%) and unpleasant odours (3.4%) as compared to the control condition (6.5%, *p*-values < 0.001), while no differences were found between the former (*p* = 1.000).

#### Faster Go responses when exposed to an odour as compared to clean air regardless of valence

A significant main effect of valence on the RTs of correct Go responses was found (Fig. 5, panel c), χ^2^(2) = 180.3, *p* < 0.001, AIC_RL_ > 100. Multiple comparisons revealed slower responses in the control condition (350 ± 56 ms) as when trials were primed with pleasant (330 ± 51 ms, *p* < 0.001) and unpleasant odours (331 ± 52 ms, *p* < 0.001), whereas no differences were found between the pleasant and unpleasant conditions (*p* = 1.000).

## Discussion

In two experiments, we examined the effect of task-irrelevant pleasant (orange) and unpleasant (trimethyloxazole and hexenol) odours on response inhibition. In Experiment 1, our findings line up with our hypotheses as more failed-to-No-Go responses, less failed-to-Go responses and faster correct Go responses emerged when trials were preceded by a pleasant odour as compared to a control condition (clean air). For RTs only, both pleasant and unpleasant odour contexts facilitated response readiness in comparison to the control condition, yet with the former presenting the shortest latencies. In other words, emotionally-charged odours increase response readiness with respect to the control condition, irrespective of their valence. In Experiment 2, we found that the rate of failed-to-No-Go responses was not affected by the odour stimuli, whereas more accurate and faster Go responses were revealed when participants were presented with pleasant and unpleasant odours as compared to the control condition, regardless of valence.

### Orthonasal pleasant odours impair response inhibition

Our results on action withholding under positive emotional context (induced by orange odour via the olfactory pathway) are in line with previous findings in Go/No-Go paradigms^3,10^. Contextual odours significantly modulated response inhibition, as participants found more challenging to withhold their responses towards visual targets in positively (but not negatively) valenced contexts vs. a control condition^3,10^. We hypothesised, in line with Chiu *et al*.^3^, that a pleasant stimulus motivates the participant to execute an action, reducing the ability to inhibiting that very same action when initiated. In the words of the spillover theory, the motivation to act elicited by the pleasant stimulus exudes (or spills over) into the motor system (a key node of the inhibitory network^33,41,42^), and as a result, inappropriate Go responses are produced in response to No-Go demands. In the study of Chiu *et al*.^3^, this “spillover” is also revealed as a modulation in motor excitability, measured as motor evoked potentials (MEPs) following single pulse TMS on the hand sector of the primary motor cortex. Following the presentation of an appetitive (vs. neutral) cue, motor excitability is increased whereas it is reduced following an aversive (vs. neutral) cue. Previous evidence demonstrate that orthonasal (pleasant) odours potentiate the motor excitability of primary motor cortex^43^, and that they activate primary (piriform) and secondary (orbitofrontal) olfactory cortices as well as a wide range of areas included in the action observation system^44^. In lack of direct evidence on the neural underpinnings of odour-modulated response inhibition, we speculate that the orbitofrontal cortex which also is involved in value-guided behaviour^45^ mediates the spillover of motivational information to the motor system though the striatum, which integrates information across reward, cognitive, and motor functions^46^.

Alternatively, Yang *et al*.^47^ suggested that affective stimuli interfere with response inhibition due to a perceptual prioritisation of emotional content when action withholding is required, as revealed by larger N170 amplitudes [i.e. an event-related potential (ERP) component used as an index of perceptual processing] elicited by emotional vs. neutral stimuli and in No-Go vs. Go trials. Albert *et al*.^10^ found larger frontocentral No-Go P3 amplitudes (i.e., an ERP component used as a robust indicator of response inhibition) and a stronger activation of the anterior cingulate cortex (i.e., a crucial area in the interaction of inhibition and emotion) in positively (vs. negatively) valenced contexts, revealing a greater mobilisation of inhibitory resources as context valence (rather than arousal) was more positive. These results are opposed to the behavioural findings obtained with the stop signal paradigm by Verbruggen and De Houwer^17^, who proposed arousal (and not valence) as responsible for the modulation of response inhibition under emotional context (i.e., greater interference at higher arousal levels regardless of valence). Critically, although both Go/No-Go and stop signal paradigms engage the active suppression of motor actions and represent measures of reactive response inhibition (i.e., triggered as a consequence of unexpected changes in the environment or internal state^48,49^), they are considered to assess different inhibitory processes: action withholding by the Go/No-Go task (i.e., the process of restraining prepared but uninitiated responses), and action cancellation by the stop signal task (i.e., the process stopping speeded already initiated actions^49,50^). Thus, we believe that our findings support the idea that action withholding is affected by the valence of isointense odour stimuli cuing the action, whereas further research is needed to evaluate the effects of valence on action cancellation.

### Valence does not affect the ability to anticipate response inhibition

It should be noted that the rate of failed-to-No-Go responses was significantly affected by the number of preceding Go trials before a No-Go trial (see Supplementary Results 7) in Experiment 1. In line with previous studies^24,51^, participants made more mistakes as the number of preceding Go trials increased, indicating that the trend to respond became stronger after each Go response, thus interfering with response inhibition. However, the valence effect was independent from the number of preceding Go trials before as the interaction between these factors was not statistically significant.

In Experiment 1, we have shown that emotional contexts tended to produce more inaccurate withholding (higher rates of failed-to-No-Go responses) than the control condition (i.e., clean air). However, this is not the case in Experiment 2. This latter result could be explained assuming that the prioritisation of emotional processing (which interferes with response inhibition) might take place exclusively when the level of threat or emotional content is highly significant^47^. Thus, it could be argued that the odours employed in Experiment 2, given the context, were not perceived as salient enough.

### Orthonasal pleasant odours facilitate response readiness more than unpleasant ones

We found that participants were more accurate and faster in Go trials under the pleasant and unpleasant odour context as compared to the control, especially in the former. This is in line with the findings of Yang *et al*.^47^ who indicated that greater attentional resources are allocated on emotional stimuli, as shown by faster RTs (and larger P3 amplitudes) with respect to neutral stimuli. The fact that we found faster Go responses under the pleasant odour context is in line with the notion that pleasant stimuli trigger action^3,5^. The same pattern was reported by Albert *et al*.^10^, suggesting that positive affect enhances thought and action repertoires (e.g., Go performance) by compromising inhibitory mechanisms. This is particularly relevant when we refer to odour stimuli as they are more likely to capture attention when they are particularly pleasant and strong^30^. In our results, this is not only evident in a response bias in the latencies, but also in the rate of failed-to-Go responses which was reduced in the pleasant condition as compared to the control one, indicating that more attentional failures in the latter.

Our findings of faster Go responses and less accurate action withholding are in line with previous evidence including task-irrelevant chemosensory stimuli (i.e., flavours in^3^) and in contrast with previous research showing that visual emotional stimuli affect both response readiness and response inhibition only when they are task-relevant^6^. This supports the idea of a modality-specific effect of emotional cues on response readiness and inhibition, with odours triggering stronger emotional responses than visual stimuli^28^.

The findings from Experiment 1 extend the scant literature on the impact of odour stimuli on response inhibition, and they overcome some of their most relevant limitations (i) by employing a computer-controlled olfactometer to potentiate the speed and accuracy of experimental designs^52^ and (ii) by including isointense pleasant and unpleasant odours, as well as a control condition, source of previous mixed results^37–40^.

### Odour valence does not affect interference control

In Experiment 2, which was aimed at investigating the effect of valence on interference control with a slow-paced Go/No-Go task, we found that the rate of failed-to-No-Go responses was not affected by the valence of the odour, whereas both pleasant and unpleasant odours were associated with more accurate and faster Go responses than in the control condition.

We anticipated a failure of the odour valence in modulating the rate of failed-to-No-Go responses due to task-specific parameters that lead to a floor effect, as reflected in equivalent low rate of failed responses across conditions (<5%), in line with previous behavioural results from slow-paced designs^18,19^. This represents a 4–5-fold reduction in the number of responses as compared to Experiment 1 (in contrast with the ~2-fold reduction for Failed-to-Go trials). In this regard, both temporal constraints and ratio of No-Go trials play a significant role in the effective capture of inhibition-related activity in the Go/No-Go task^7,8^. For instance, Wessel^7^ revealed that Go/No-Go paradigms including equiprobable proportions of Go/No-Go trials and slow-paced designs, engage less frontocentral activity associated to response inhibition, thus, involving fewer inhibitory demand. In this regard, although Experiment 2 included more No-Go trials (~3:1) than Experiment 1 (5:1), the task was slow-paced (average ITI = 5.5 s), which lead to a lower pressure to respond. Along the same lines, Zamorano *et al*.^9^ observed that N2 amplitude (i.e., inhibition-related ERP component) in No-Go trials was higher in fast-paced vs. slow-paced designs, reflecting greater inhibitory requirements by the former. These authors highlighted that while fast-paced designs involve more automatic processing characteristic of reactive response inhibition (leading to faster and more inaccurate responses), slow-paced Go/No-Go tasks involve different aspects of cognitive control, such as attentional interference and decision making processes, that imply less motor inhibitory demands (leading to slower and more accurate responses). Albert *et al*.^10^ found that No-Go P3, but not N2, was modulated by emotional context, in line with the idea that the No-Go P3 represents an ERP component associated to the inhibitory process itself, whereas the N2 relates to different components of response inhibition (e.g., pre-motor inhibition, response activation and conflict monitoring). These authors indicated that emotional modulations interact particularly with the inhibitory process itself rather than other aspects of response inhibition, as the ones involved in slow-paced designs.

It should be noted that due to technical problems, we did not analyse the effect of preceding Go trials on the rate of failed-to-No-Go responses in Experiment 2. However, due a floor effect we would expect that a possible significant effect of this manipulation (i.e., 1, 3, 5 or 7 preceding Go trials before a No-Go trial) would be independent of the olfactory manipulation, which did not modulate the error rate in No-Go trials.

### Under reduced time pressure, task-irrelevant odours speed up motor responses irrespective of valence

As expected, more accurate and faster Go responses where revealed when Go trials were primed by an odour as compared to the control condition. We anticipated no differences regarding valence due to the low cognitive load and motivation pressure of the task (i.e., not accounting for action biases by pleasant contexts). As an important index of behavioural execution^5,53^, faster latencies indicate that participant’s responses were biased by both pleasant and unpleasant odours, in line with previous research showing that faster responses where elicited by emotional (vs. neutral) stimuli^47,54^. As for the rate of failed-to-Go responses, participants were more prone to commit errors in the control condition. Thus, lapses of attention were less frequent under emotional contexts. In this respect, results regarding Go responses support the idea that emotional (vs. non-emotional) stimuli capture more attention^47^.

## Conclusions

In summary, we revealed that pleasant (but not unpleasant) task-irrelevant odours modulate response inhibition, more specifically, impairing the withholding of prepotent motor responses in the Go/No-Go task. The inclusion of orthonasal olfactory cues into Go/No-Go paradigms represents a more suitable emotional manipulation than visual cues as they are strong and fast triggers of emotion, with the potential of acting in lack of awareness. In line with the spillover theory, pleasant stimuli biased behaviours towards action. When No-Go cues were presented, this trend was revealed in more inaccurate action withholding. When Go cues were presented, more accurate and faster responses (as compared to the unpleasant and control conditions) were elicited. These results support the idea that more inhibitory resources are required under positively valenced contexts. It should be noted that the unpleasant odour context also facilitated response readiness as shown by faster Go responses as compared to the control condition, in line with the idea that more attentional resources are allocated on emotional stimuli. Importantly, the effect of emotional contexts was revealed only when the Go/No-Go task was fast paced, as it was more capable of evoking a trend to elicit overt responses by involving more inhibitory demands as compared to slow-paced designs. To further disentangle this aspect, future research should identify paradigms able to distinguish between performance in motor and attentional inhibition tasks that are free of floor effects. Moreover, future research should address whether this effect is also revealed when the emotional context induced via olfactory stimuli is task-relevant. Despite all odours included in this study were presented at supra-threshold levels, it is possible that the unpleasant odour was not relevant or salient enough to provoke an effect on response inhibition in the slow-paced design. Future research could address this issue by including odours of different intensities or inducing aversive responses towards specific odours via conditioning. Additionally, the generalisability of the present results can be tested by including different pleasant odours, even though the evidence hereby presented on unpleasant odours suggests that these results should not be odour specific. Despite our observation of the orange odour-induced modulation in response readiness, which is not present with any of the unpleasant odours tested, the generalisability of the odour pleasantness-dependent effect will be explored in future studies with several different categories of pleasant odours such as food, non-food, floral, sandalwood or balsamic odours. On the other hand, a limitation for the generalisation of our findings is the possibility that the impairment of response inhibition relies in specific properties of the pleasant odour selected for this study (i.e., orange). Moreover, futures studies might include psychophysiological measures (e.g., skin conductance response) to better account for the arousal aspect of processing of odour cues.

## Materials and Methods

### Participants

#### Experiment 1

Thirty-one healthy participants were recruited by convenience sampling (16 women, mean age = 26.1 ± 4.1 years old, age range 19–34 years old). Power analysis (G*Power^55^) for a medium effect size at power = 0.90 and α = 0.05 suggested a sample size of twenty-nine; we slightly oversampled to prevent possible technical failures. An initial screening was carried out through an online survey to account for the following exclusion criteria: past head trauma with loss of consciousness, ex-smoker for more than six months, drinking habits (more than five times a week), anxiety (below cut off score of 43 at the State-Trait Inventory for Cognitive and Somatic Anxiety^56^), and depression (below cut off score of 17 at the Beck Depression Inventory-II^57^). All participants were right-handed (assessed by the Edinburgh Handedness Inventory^58^ adopting a cut off above 60), had normal olfactory function (cut off ≥10 at the Identification subtest of the Sniffin’ Sticks test; Burghart®, Wedel, Germany^59^), and self-reported normal or corrected to normal vision. Each participant received 8 Euro after completing the experiment.

#### Experiment 2

Twenty-nine healthy participants were recruited by convenience sampling (24 women, mean age = 23.5 ± 3.2 years old, age range 19–33 years old). Inclusion criteria were the same as in Experiment 1. None of the participants that took part in Experiment 1 were included in this sample. Participants received 12 Euro as compensation for completing the experiment.

All participants from Experiment 1 and Experiment 2 were informed about the experimental procedures and gave their written consent. Participants were debriefed about the purpose of the study at the end of the experiments. Participants were instructed to avoid ingestion of anything but water from 1 h prior to testing, and to avoid wearing any scented products on the day of testing. All procedures in both Experiment 1 and Experiment 2 (including the fact that participants remained naïve about the purpose of the study until they completed the experiment) were approved by the local Institutional Review Board (International School for Advanced Studies, SISSA) and were in compliance with the Declaration of Helsinki^60^.

### Apparatus and stimuli

#### Experiment 1

Three odours, all diluted with propylene glycol, were used as olfactory cues: orange (30% v/v, Givaudan), methional (80% v/v, Sigma-Aldrich), 2,4,5-trimethyloxazole (0.5% v/v, Sigma-Aldrich), while clean air (over propylene glycol) was included as control condition. These concentrations were selected based on a pilot study that aimed to identify pleasant (orange), neutral (methional, an onion- and meat-like odour) and unpleasant (trimethyloxazole, a burnt nutty odour) odours that were isointense (see Supplementary Results 1); participants that took part in the pilot study were not considered in the experiment. However, in Experiment 1 participants rated methional as equally unpleasant as trimethyloxazole, but less intense than the isointense orange and trimethyloxazole (see Supplementary Results 2). Thus, we removed the methional trials and we refer to orange as the pleasant solution, trimethyloxazole as the unpleasant solution and to clean air as the control stimulus. We report the main analyses for methional in the Supplementary Results 3; importantly, all participants but one followed the expected valence pattern when rating the conditions. The analyses with and without such participant do not differ, therefore we included them in the analysis. The odour solutions were stored in sanitised glass jars (3 mL solution in straight-sided glass 4 oz jars, Uline, Pleasant Prairie, WI, USA), delivered using a customised olfactometer (Sniff-0, CyNexo, Udine, Italy, http://www.cynexo.com). A constant flowing air stream (0.5 L/min) was maintained across the whole experiment, while odour stimuli were presented at a flow of 3 L/min (e.g.^29^) via cannulas covered with custom-made nose-pieces, birhinally placed in the nasal cavities. The presented odour compounds exceeded detection threshold, as verified during debriefing with the participants, all of whom were able to smell each odour. Following Littman and Takács^53^, the Go/No-Go cues included in the task consisted of either a white filled circle or square (2.5 × 2.5 cm). Visual targets, fixation crosses (1.5 × 1.5 cm), visual analogue scales (VAS) and textual information (i.e., task instructions and feedbacks) were presented on a 19″ LCD monitor (Samsung SyncMaster 940 T, 1280 × 1024 resolution) against a black background at an approximated distance of 50 cm from the participant.

#### Experiment 2

Three odours (Givaudan) diluted with propylene glycol were used as olfactory stimuli: orange (20% v/v), hexenol (5% v/v), civet (0.2% v/v); clean air was used as control condition. The concentrations were selected based on a pilot study (see Supplementary Results 4). None of the participants from the pilot study took part in the experiment. We changed the solutions from Experiment 1 (methional and trimethyloxazole, specifically) in order to identify isointense pleasant (orange), neutral (hexenol, intense grass odour) and unpleasant (civet, a musk-like oily odour) odours, and to determine whether the lack of a significant effect with the unpleasant odour depended on the particular solution presented. Nevertheless, it should be noted that in Experiment 2 the civet odour was perceived as equally unpleasant as hexenol but less intense than the other two isointense odours (see Supplementary Results 5). Thus, the odour stimuli were categorised as follows according to their valence: pleasant (orange), unpleasant (hexenol), and control (clean air), removing the civet from the analyses (but are reported in the Supplementary Results 6). As in Experiment 1, all participants (but 4) followed this pattern. Following their ratings, we reversed the pleasantness attribution. Results including or excluding them are equivalent, therefore we maintained them within the database. All the remaining aspects were the same as in Experiment 1.

### Procedure

#### Experiment 1

The main task consisted of an olfactory Go/No-Go task that included the delivery of either an odour or clean air prior to the presentation of a visual target (i.e., a circle or a square). Each trial started with a black screen and the delivery of clean air for 500 ms (Fig. 1, panel a), followed by the onset of a white fixation cross in the centre of the screen that indicated to the participants to sniff once. During fixation (sniff cue), one of the three olfactory stimuli was presented during 1,000 ms. In line with previous literature, this duration is considered sufficient to allow for the detection of an odour in a single sniff (>750 ms used in^61^; see also^62^). When the target appeared at the centre of the screen, clean air was delivered. Clean air was presented at a flow-rate 6-fold higher than the constant flow. Whether the participant answered or not, the target remained on screen for 500 ms^53^. After target offset, the screen remained black and clean air was delivered until the end of the trial to clean any residuals of previously delivered odours. The average ITI ranged from 400 to 600 ms (500 ms average). Each trial lasted 2.5 s on average. The task included eight experimental blocks that were presented in a different order following a Latin square design. The blocks were composed of 100 trials each (25 per odour condition), with a 5:1 ratio of Go/No-Go trials within each block^5^. Participants were instructed to respond by pressing the spacebar of a keyboard with their right index finger when a circle (Go cue) was presented and withhold their response when a square (No-Go cue) appeared. Fifteen participants received the opposite mapping instructions (i.e., square as Go cue and circle as No-Go cue) and data from all participants were collapsed. Participants were instructed to respond while the target was on screen (500 ms response deadline). Trials were presented following a pseudo-randomised order in which No-Go trials were preceded by equiprobable 1, 3, 5 or 7 Go trials^9^. In order to avoid habituation effects, the repetition of a given odour was always preceded by at least two different odours in between. This procedure included continuous changes in the olfactory environment which increase the probability of detecting each odour, even at short ITI^63^. The rates of failed trials and mean RTs were presented for 10 s as feedback on screen after each block^64^, allowing the experimenter to monitor participants’ performance throughout the experiment. For the sake of brevity, we move the details on the breathing training, familiarisation block and rating procedure (i.e., perceive pleasantness and intensity of the odour conditions) to the Supplementary Methods. Participants were reminded to breathe as trained and to respond as accurately and as fast as possible throughout the experiment. The tasks were programmed and presented using the E-Prime 2.0 software^65^. The experiment lasted about 60 minutes.

#### Experiment 2

Participants performed the Go/No-Go task described in Experiment 1 including the following modifications: each trial started with a black screen for 500 ms (Fig. 1, panel b) as clean air was delivered, followed by the onset of a white fixation cross in the centre of the screen for 500 ms which indicated to participants that they had to prepare to smell. With the onset of the fixation cross, clean air was switched off and one of the olfactory stimuli was delivered. Then, the white cross turned red and remained on screen for 750 ms indicating to the participants to sniff once (sniff cue). This change was made to maximise the accuracy of the odour detection (e.g., red is a more salient colour^66^) considering the fewer temporal constraints intrinsic to slow-paced designs. In total, the odour stimulus was delivered during 1.25 s (fixation + sniff cue). After this, the target was presented at the centre of the screen as clean air was delivered once again. The target remained on screen for 500 ms regardless of participant’s response. The screen remained black after target offset until the end of the trial. The ITI ranged from 5 to 6 s (5.5 s average). A single trial lasted 7.75 s on average. The task included four experimental blocks presented following a Latin square design. Each block was composed of 80 trials (20 per odour condition), with a ~3:1 ratio of Go/No-Go trials^53^. All the other aspects were the same as in Experiment 1. The experiment lasted about 90 minutes.

### Statistical analyses

Data were analysed using *R*^67^ considering the following dependent variables: perceived pleasantness and intensity of the olfactory stimuli (i.e., VAS ratings), rates of failed-to-No-Go (i.e., No-Go trials in which participants pressed the spacebar) and failed-to-Go responses (i.e., Go trials in which participants did not press the spacebar), and RTs of correct Go response (i.e., time elapsed from target presentation to the participant’s response on correct Go trials). Linear mixed-effects models (LME) were computed for intensity, pleasantness and RTs, whereas generalised mixed-effects models (GLME) with binomial link function were computed for the rates of failed-to-Go and failed-to-No-Go responses. Mixed-effects modelling have a series of advantages in front of more conventional approaches (e.g., repeated-measures ANOVA). It allows to take into account simultaneously all the potential factors that might contribute to the explanation of the data. Moreover, it provides enhanced statistical power for designs including repeated-measures (as in the present study) and allows to better deal with unbalanced data sets^68^. For the analyses of RTs, trials exceeding 2.5 standard deviations were excluded^64^ (1.8% of the data of Experiment 1 and Experiment 2). In all LME and GLME models, participants were included as random effect. For pleasantness and intensity models, the categorical variables odour (i.e., control vs. orange vs. trimethyloxazole) and cycle (i.e., times at which the participants rated the odour conditions) were added as a fixed effects, the latter in order to verify that the perception of the odour conditions was stable throughout the experiment. For the rates of failed-to-No-Go and failed-to-Go responses and RTs models, the categorical variable valence (i.e., pleasant vs. unpleasant vs. control) was added as a fixed effect. For the rates failed-to-Go responses only, the categorical variable preceding Go trials (1, 3, 5 and 7) was included. However, due to technical problems the recording of this manipulation was only possible in Experiment 1. The results regarding the effect of preceding Go trials are presented in the Supplementary Information (see Supplementary Results 7). Following a model comparison approach^69^, models including a given factor and models without it were contrasted using the Akaike information criterion (AIC), based on which the model with the lowest AIC was considered the best fitting model^70^ (e.g., the LME model including valence as fixed factor was compared against the null LME model without the valence factor to determine the effect of this variable on RTs; this procedure was repeated for all variables included in the analyses as well as their interactions). The difference between AICs across models was calculated to determine the relative likelihood of a given model compared to another [AIC_RL_ = exp(ΔAIC/2), e.g.^71^). The corresponding *p*-values were extracted from likelihood ratio tests. Generalised linear hypothesis testing and Tukey’s HSD were used to perform multiple comparisons of means on the significant effects, including Bonferroni correction and with the *α* level set at 0.05. Optimisers for the control of mixed model fitting (i.e., bobyqa) were employed in GLME models that failed to converge when analysing failed-to-No-Go responses. For LME models the mean and standard deviation of the corresponding conditions were estimated for reporting the results. For GLME model the percents of failed trials per condition were reported. To allow for replicability, all the *R* packages that have been used are reported in the Supplementary Methods.

## Supplementary information

Supplementary Information

## Data Availability

The datasets generated during and/or analysed during the current study are available in the Open Science Framework (OSF) repository accessible at https://osf.io/sh4tp/?view_only=9447f311067b47ad95e88188b610f729.
