# Supplementary material for: Task-irrelevant odours affect both response inhibition and response readiness in fast-paced Go/No-Go task: the case of valence
Source: Sci Rep. 2019 Dec 18;9:19329. doi: 10.1038/s41598-019-55977-z (PMC6920346; doi:10.1038/s41598-019-55977-z)
Supplement: Supplementary file 1 — Supplementary Information [file 41598_2019_55977_MOESM1_ESM.doc]

**Task-irrelevant odours affect both response inhibition and response readiness in fast-paced Go/No-Go task: the case of valence**

Javier Albayay^*^, Umberto Castiello, and Valentina Parma

*E-mail: javier.albayay@gmail.com

**Supplementary Information**

***Supplementary Results 1***

***Results from the pilot study for Experiment 1.*** In order to identify pleasant, neutral and unpleasant olfactory stimuli to be used in Experiment 1, fifteen participants (6 women, mean age = 28.2 ± 3.1 years old, age range 22-34 years old) were presented with the following odours diluted with propylene glycol: orange (30% v/v, Givaudan), methional (80% v/v, Sigma-Aldrich), and 2,4,5-trimethyloxazole (0.5% v/v, Sigma-Aldrich). Clean air (over propylene glycol) was presented as control condition. Odours and clean air were delivered separately through an olfactometer (Sniff-0, CyNexo, Udine, Italy, http://www.cynexo.com) and were rated on pleasantness and intensity (Supplementary Fig. S1) in two visual analogue scales (VAS) ranging from 0 (not at all) to 100 (very much). Each rating trial started with a white fixation cross (1.5 × 1.5 cm) presented for two seconds followed by the presentation of the VAS. The VAS remained on screen until the participant rated the given olfactory stimuli using a computer mouse.

A significant main effect of odour on pleasantness was evident, χ^2^(3) = 252.22, *p* < 0.001, AIC_RL_ > 100. The orange odour (84.3 ± 11.6) was perceived as more pleasant as compared to the other conditions (all *p*-values < 0.001), trimethyloxazole (17.4 ± 17.5) was rated as the most unpleasant odour (all *p*-values < 0.001), and methional (42.2 ± 13.2) and clean air (48.6 ± 12.2) were equally rated as neutral (*p* = 0.172). As for the intensity rating, a significant main effect of odour was retrieved, χ^2^(3) = 175.76, *p* < 0.001, AIC_RL_ > 100. All odorous conditions were rated as isointense: orange (59.5 ± 21.1) vs. methional (53 ± 15.7, *p* = 0.223), orange vs. trimethyloxazole (60.1 ± 23.2, *p* = 1.000), methional vs. trimethyloxazole (*p* = 0.137) whereas clean air (13.4 ± 17.5) was significantly less intense as compared to the three odours (all *p*-values < 0.001).


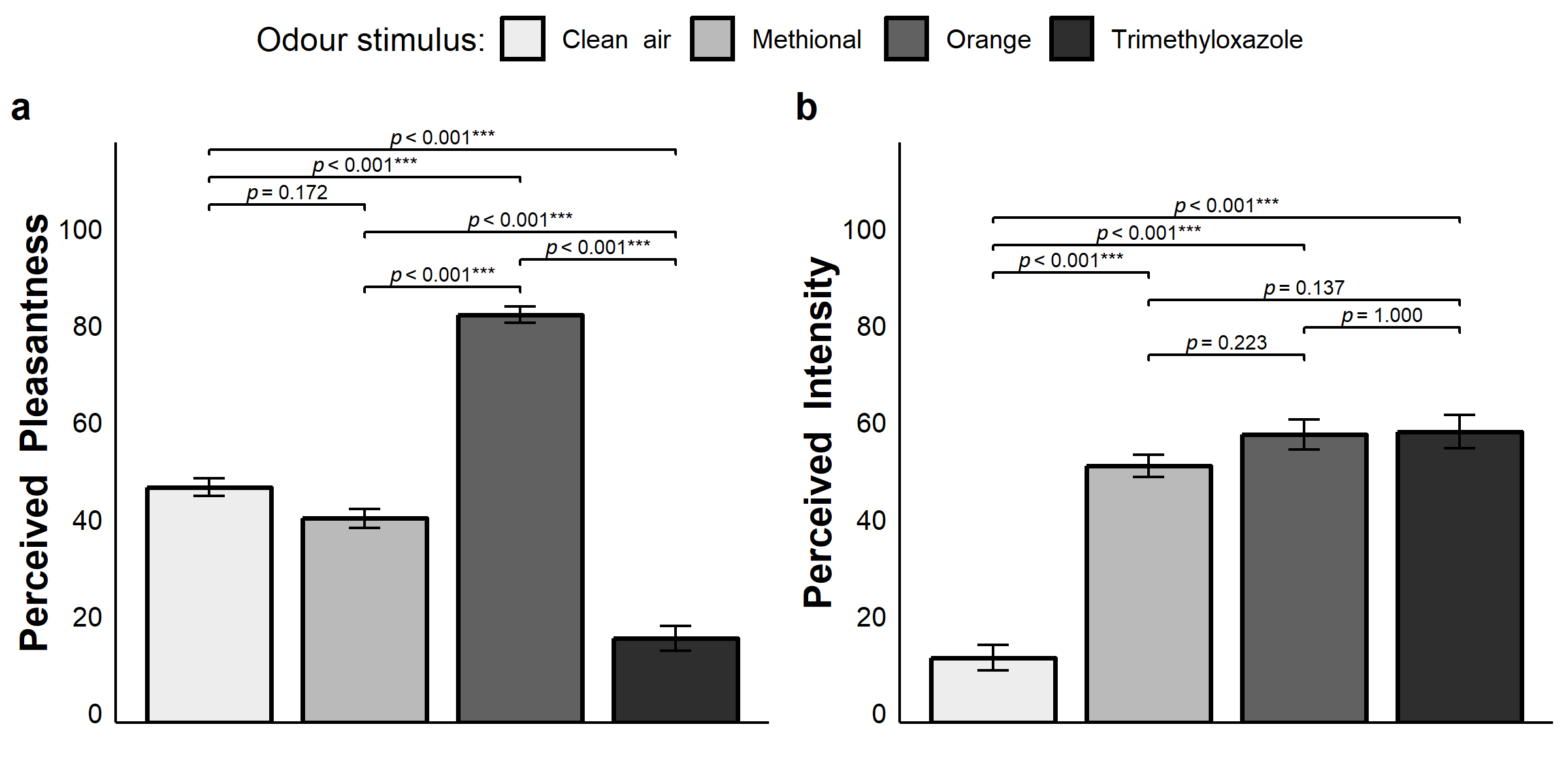


**Supplementary** **Figure S1.** Results from the pilot study for Experiment 1. (**a**) Perceived pleasantness of the odour stimuli. (**b**) Perceived intensity of the odour stimuli. Error bars represent standard error of the mean.

***Supplementary Results 2***

***Odour ratings of Experiment 1 without excluding the methional odour.*** In Experiment 1, methional was not considered in the analyses given that it was perceived as less intense as compared to the other odours. Here we present the results of the perceived pleasantness and intensity of Experiment 1 without excluding this odour condition (Supplementary Fig. S2).

With respect to pleasantness, the main effect of odour was significant, χ^2^(3) = 755.38, *p* < 0.001, AIC_RL_ > 100, whereas neither the main effect of cycle, χ^2^(8) = 1.638, *p* = 0.990, AIC_RL_ = 0.447, nor the interaction odour × cycle, χ^2^(24) = 17.142, *p* = 0.842, AIC_RL_ < 0.001, reached significance. The orange odour (73.3 ± 18.1) was perceived as more pleasant as compared to the other conditions (all *p*-values < 0.001), whereas trimethyloxazole (29.9 ± 24.5) and methional (29.3 ± 22.7) were rated as equally unpleasant (*p* = 1.000) as compared to clean air (45.2 ± 13.5, *p*-values < 0.001). On the other hand, a significant main effect of odour was revealed on intensity, χ^2^(3) = 1015.7, *p* < 0.001, AIC_RL_ > 100, while the main effect of cycle, χ^2^(8) = 14.39, *p* = 0.072, AIC_RL_ = 0.447, and the interaction odour × cycle, χ^2^(24) = 12.638, *p* = 0.972, AIC_RL_ < 0.001, were not significant. Clean air (9.4 ± 14.6) was rated as less intense as compared to the odours (all *p*-values < 0.001). Only orange (62.5 ± 24.2) and trimethyloxazole (66 ± 26) were perceived as isointense (*p* = 0.172), whereas methional (56.8 ± 26.9) was rated as less intense as compared to both orange (*p* = 0.002) and trimethyloxazole (*p* < 0.001).


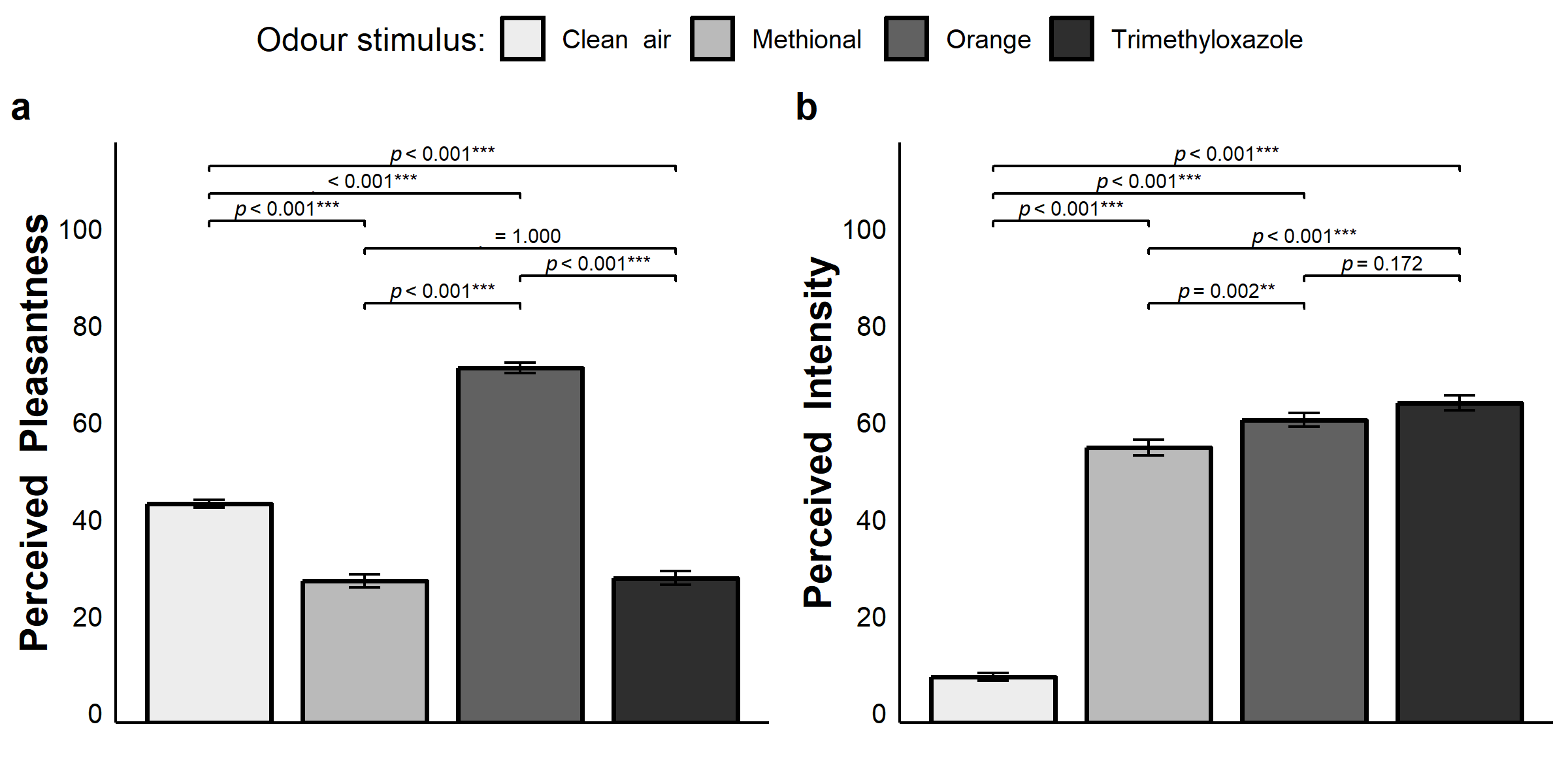


**Supplementary** **Figure S2.** Odour ratings of Experiment 1 without excluding the methional odour. Perceived pleasantness of the odour stimuli. (**b**) Perceived intensity of the odour stimuli. Error bars represent standard error of the mean.

***Supplementary Results 3***

***Results of Experiment 1 as a function of odour.*** Here we present the results of Experiment 1 (i.e., rates of failed-to-No-Go responses, failed-to-Go responses and RTs of correct Go response) as a function of odour, without excluding methional (Supplementary Fig. S3).

A significant effect of odour on failed-to-No-Go responses was revealed, χ^2^(3) = 11.494, *p* = 0.009, AIC_RL_ = 15.592. Participants were less accurate when clean air (16%) was presented as when the orange odour (20.1%, *p* = 0.005) was delivered, whereas no other comparison reached significance: clean air vs. trimethyloxazole (19.1%, *p* = 0.175), clean air vs. methional (18.5%, *p* = 0.486), orange vs. trimethyloxazole (*p* = 1.000), orange vs. methional (*p* = 0.641), trimethyloxazole vs. methional (*p* = 1.000). As for failed-to-Go responses, a significant main effect of odour emerged, χ^2^(3) = 9.219, *p* = 0.026, AIC_RL_ = 5. Multiple comparisons revealed marginal differences between clean air (6.6%) and methional (5.4%, *p* = 0.054), while other comparisons were not significant: clean air vs. orange (5.6%, *p* = 0.206), clean air vs. trimethyloxazole (5.4%, *p* = 0.062), orange vs. methional (*p* = 1.000), orange vs. trimethyloxazole (*p* = 1.000), trimethyloxazole vs. methional (*p* = 1.000). Finally, a significant main effect of odour was retrieved on RTs, χ^2^(3) = 195.02, *p* < 0.001, AIC_RL_ > 100. Slower responses were elicited when clean air (334.4 ± 59.8 ms) was presented as compared to the delivery of odours (all *p*-values < 0.001). Comparisons among the odours showed faster Go responses for orange (329.5 ± 58.1 ms) as compared to both trimethyloxazole (334.5 ± 58.9 ms, *p* < 0.001) and methional (334.3 ± 58.9 ms, *p* < 0.001), whereas trimethyloxazole and methional did not differ significantly (*p* = 1.000).


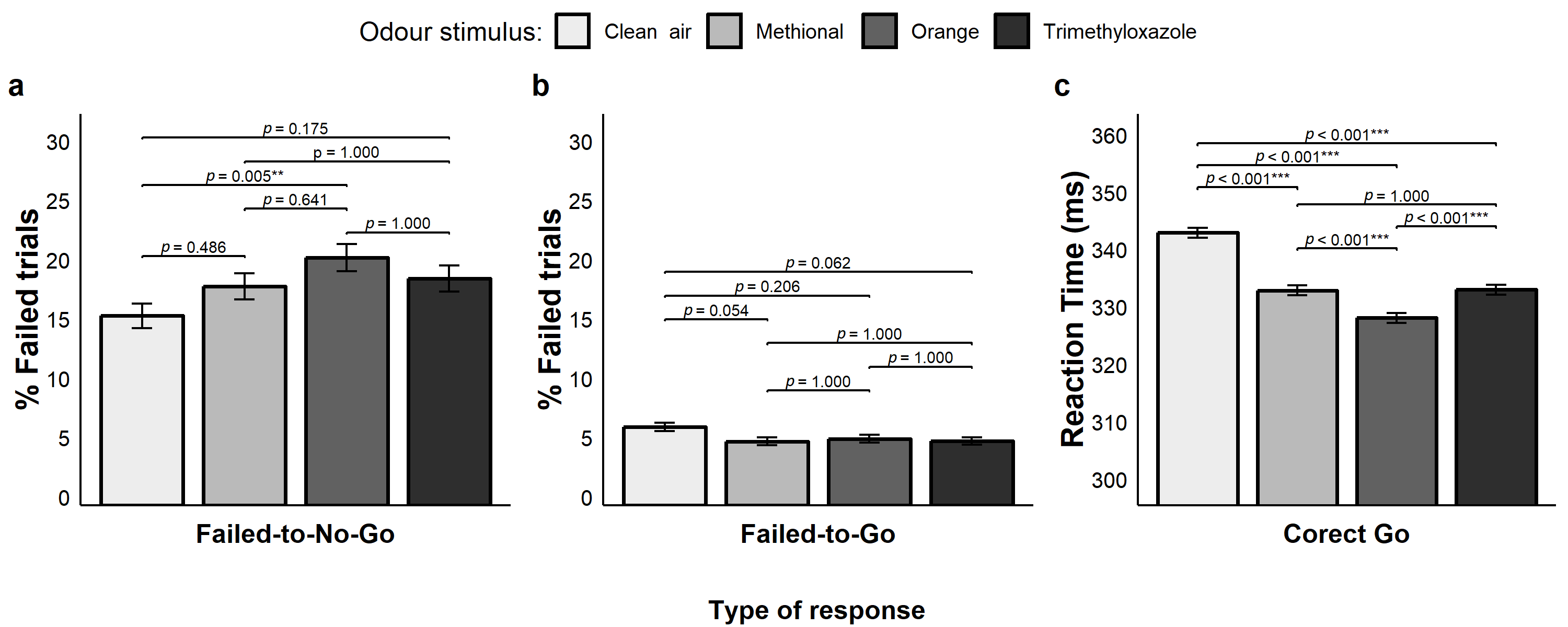


**Supplementary** **Figure S3.** Results of Experiment 1 as a function of odour. (**a**) Rates of failed-to-No-Go responses. (**b**) Rates of failed-to-Go responses. (**c**) Reaction times of correct Go responses. Error bars represent standard error of the mean.

***Supplementary Results 4***

***Results from the pilot study for Experiment 2.*** To identify pleasant, neutral and unpleasant olfactory stimuli to be included in Experiment 2, twenty-one participants (13 women) were presented with the following odours (Givaudan) diluted with propylene glycol: orange (20% v/v), hexenol (5% v/v), civet (0.2% v/v). Odours were presented by the experimenter using sanitized glass jars (100 mL amber bottles) containing 1 mL of the solutions. Participants smell each jar for 2 s and rated each odour on pleasantness and intensity (Supplementary Fig. S4) in a scale ranging from 0 (not at all) to 10 (very much).

The main effect of odour on pleasantness was significant, χ^2^(2) = 70.474, *p* < 0.001, AIC_RL_ > 100. The orange odour (8.5 ± 1.3) was perceived as more pleasant as compared to both hexenol (6.4 ± 1, *p* < 0.001) and civet (2.8 ± 2.3, *p* < 0.001). Civet, in turn, was rated as more unpleasant than hexenol (*p* < 0.001). On the other hand, a significant main effect of odour was not retrieved on intensity, χ^2^(2) = 3.628, *p* = 0.163, AIC_RL_ = 0.830, revealing that the three odours were perceived as isointense: orange (5.9 ± 1.7), hexenol (5 ± 1.7), and civet (5.3 ± 1.6).

**
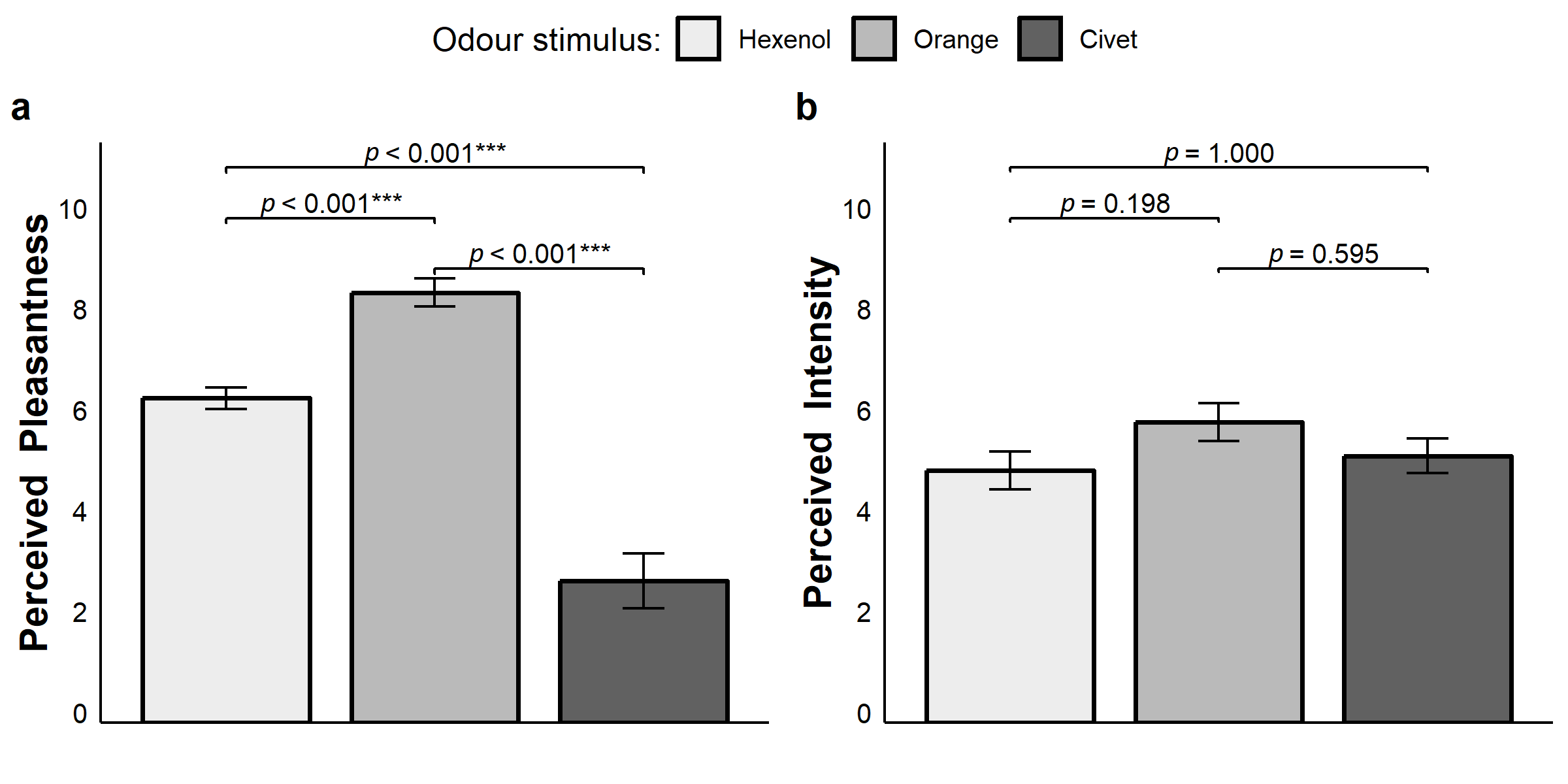
**

**Supplementary** **Figure S4.** Results from the pilot study for Experiment 2. (**a**) Perceived pleasantness of the odour stimuli. (**b**) Perceived intensity of the odour stimuli. Error bars represent standard error of the mean.

***Supplementary Results 5***

***Odour ratings of Experiment 2 without excluding the civet odour.*** In Experiment 2 we excluded the civet odour from the analyses as it was perceived as less intense as compared to the other odours. Here we present the results regarding the perceive pleasantness and intensity including civet (Supplementary Fig. S5).

A significant main effect of odour was revealed on pleasantness, χ^2^(3) = 335.7, *p* < 0.001, AIC_RL_ > 100, whereas neither the effect of cycle, χ^2^(4) = 1.134, *p* = 0.888, AIC_RL_ = 0.032, nor the interaction odour × cycle, χ^2^(12) = 5.085, *p* = 0.955, AIC_RL_ < 0.001, reached significance. The orange odour (76 ± 23.1) was more pleasant than the other stimuli (all *p*-values < 0.001), while hexenol (31.3 ± 28.3) and civet (29 ± 22.5) were perceived as equally unpleasant (*p* = 1.000) as compared to clean air (40.8 ± 18.9, *p*-values < 0.001). As for intensity, a significant main effect of odour emerged as well, χ^2^(3) = 474.55, *p* < 0.001, AIC_RL_ > 100, whereas the main effect of cycle, χ^2^(4) = 5.854, *p* = 0.210, AIC_RL_ = 0.342, and the interaction odour × cycle, χ^2^(12) = 6.857, *p* = 0.867, AIC_RL_ < 0.001, were not significant. Clean air (12.3 ± 15.3) was less intense as compared to the odour stimuli (all *p*-values < 0.001). Only orange (65.3 ± 24.5) and hexenol (70.2 ± 24.9) were perceived as isointense (*p* = 0.218), and civet (43.3 ± 25.9) was rated as less intense as compared to both of them (*p*-values < 0.001).


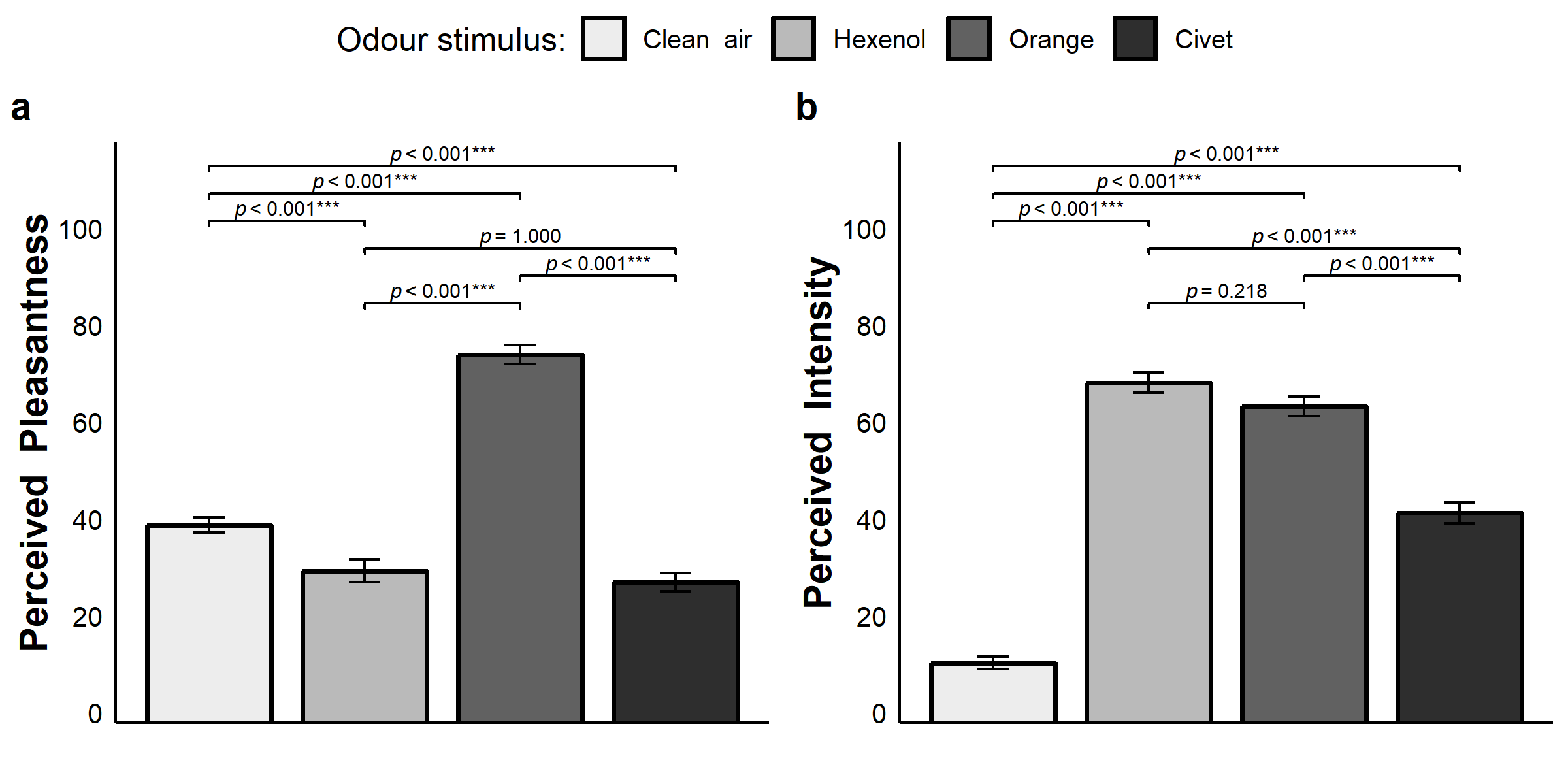


**Supplementary** **Figure S5.** Odour ratings of Experiment 2 without excluding the civet odour. Perceived pleasantness of the odour stimuli. (**b**) Perceived intensity of the odour stimuli. Error bars represent standard error of the mean.

***Supplementary Results 6***

***Results of Experiment 2 as a function of odour.*** Here we present the results of Experiment 2 (i.e., rates of failed-to-No-Go responses, failed-to-Go responses and RTs of correct Go response) for each odour stimuli, including civet (Supplementary Fig. S6).

The main effect of odour on failed-to-No-Go was not significant, χ^2^(3) = 0.071, *p* = 0.995, AIC_RL_ = 0.052 (clean air = 4.4%; orange = 4.3%; hexenol = 4.4%, civet = 4.6%). On the other hand, a significant main effect of odour was revealed on failed-to-Go, χ^2^(3) = 24.275, *p* < 0.001, AIC_RL_ > 100. Participants were less accurate when presented with clean air (6.5%) as compared to when presented with orange (3.3%, *p* < 0.001), hexenol (3.5%, *p* < 0.001) and civet (4.1%, *p* = 0.010). No other comparison reached significance: orange vs. hexenol (*p* = 1.000), orange vs. civet (*p* = 1.000), civet vs. hexenol (*p* = 1.000). Finally, the main effect of odour on RTs was significant, χ^2^(3) = 182.97, *p* < 0.001, AIC_RL_ > 100. Clean air (350.2 ± 56 ms) elicited slower Go responses as compared to all odour conditions (all *p*-values < 0.001), whereas other comparisons were not significant: orange (330.4 ± 50.8 ms) vs. hexenol (331.4 ± 52.3 ms, *p* = 1.000), orange vs. civet (333.5 ± 54.6 ms, *p* = 0.272), civet vs. hexenol (*p* = 0.865).


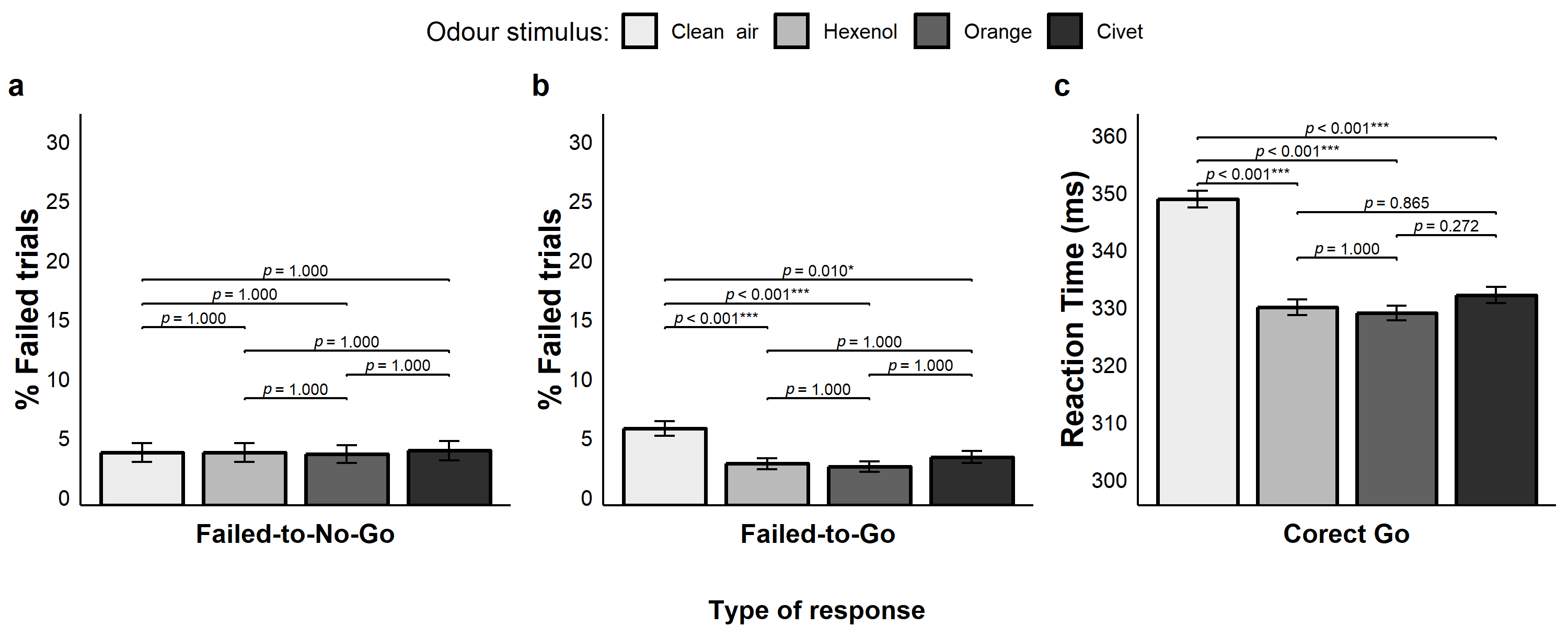


**Supplementary** **Figure S6.** Results of Experiment 2 as a function of odour. (**a**) Rates of failed-to-No-Go responses. (**b**) Rates of failed-to-Go responses. (**c**) Reaction times of correct Go responses. Error bars represent standard error of the mean.

**Supplementary Results 7**

***Rate of failed-to-No-Go responses as a function of preceding Go trials in Experiment 1*.** A significant main effect of the number of preceding Go trials on the rate of failed-to-No-Go responses was revealed (Supplementary Fig. S7), χ2(3) = 27.893, *p* < 0.001, AIC_RL_ > 100, while the interaction preceding Go trials × valence was not significant, χ2(6) = 10.514, *p* = 0.105, AIC_RL_ = 0.476. Participants were more accurate when No-Go trials were preceded by a single Go trial (14.1%) as compared to sequences of three (19.5, *p* < 0.001) and seven Go trials (22.9%, *p* < 0.001). In turn, more errors were committed after sequences of seven Go trials as compared to sequences of five Go trials (18.2%, *p* = 0. 041); other comparisons were not significant: one vs. five (p = 0.066), three vs. five (*p* = 1.000), three vs. seven (*p* = 0.308).

**
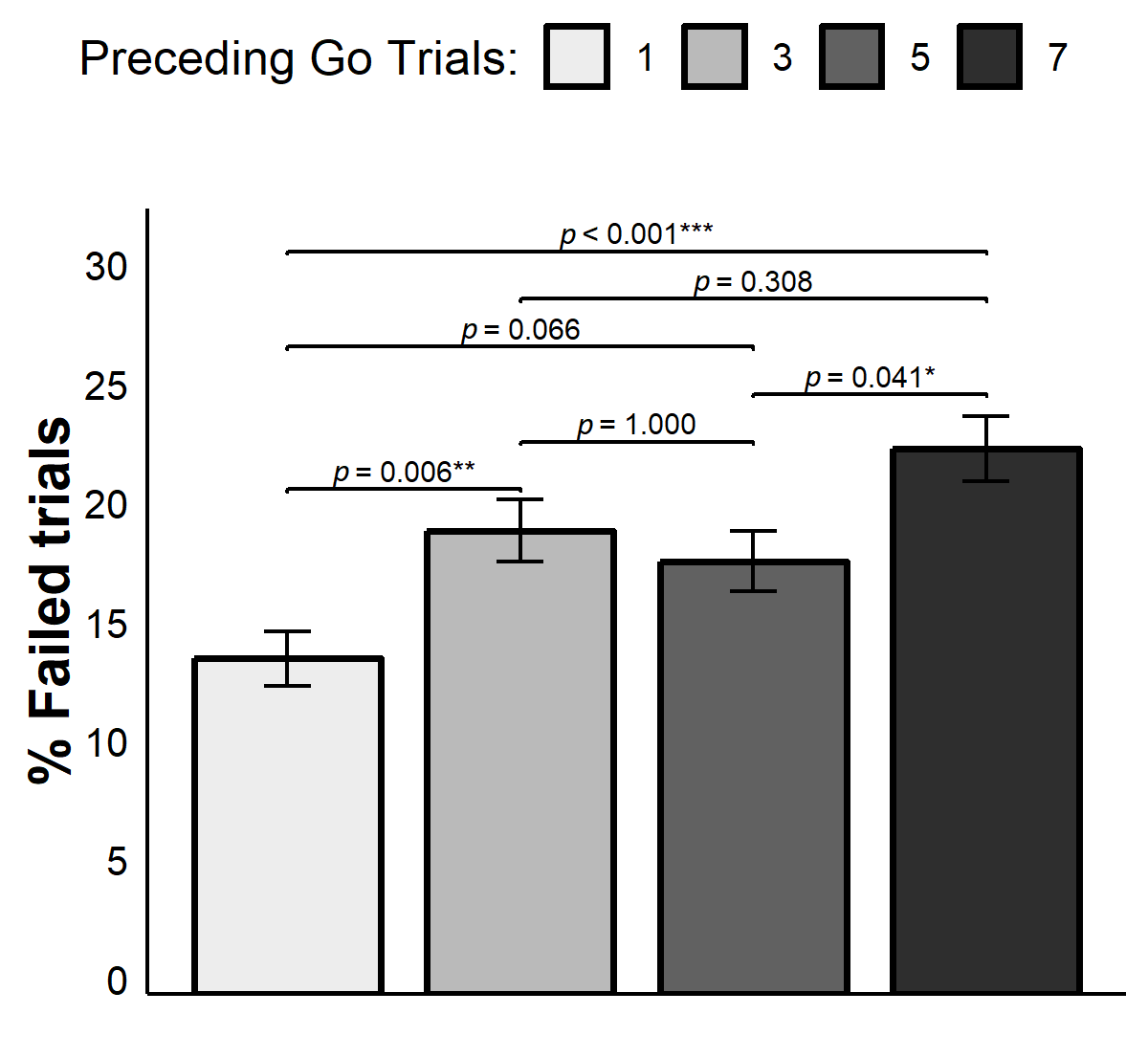
**

**Supplementary Figure S7.** Rates of failed-to-No-Go responses as a function of preceding Go trials in Experiment 1. Error bars represent standard error of the mean.

***Supplementary Methods***

***Breathing training.*** Before starting the experiment, participants performed a breathing training block composed of 30 trials that followed the same structure of the main task, but including only the delivery of clean air. The instruction was to sniff once when the fixation cross appeared on screen and then exhale until the end of the trial. The experimenter visually inspected if participants sniffed as indicated and debriefed them after each block.

***Familiarization block*.** After completing the breathing training, participants performed 30 familiarization trials). To avoid overexposure to the odours and adaptation during the practice block, only six trials were preceded by an odour while the remaining trials were preceded by clean air.

***Rating procedure*.** Once participants reached 85% accuracy in the practice block, they rated the pleasantness and intensity of the odour stimuli in two separate VAS ranging from 0 (not at all) to 100 (very much). The rating procedure was completed after each experimental block followed by a one-minute rest, with no clean air delivered to the nose.

***R packages*.** Lear mixed-effects models and generalized mixed-effects models were computed using the *nlme* (<https://cran.r-project.org/web/packages/nlme/index.html>) and *lme4* (<https://cran.r-project.org/web/packages/lme4/index.html>) packages. Generalized linear hypothesis testing and Tukey's HSD from the *multcomp* (<https://cran.r-project.org/web/packages/multcomp/index.html>) package were used to perform multiple comparisons. Figures related to data analyses were generated using the *ggplot2* (<https://cran.r-project.org/web/packages/ggplot2/index.html>) and *ggpubr* (<https://cran.r-project.org/web/packages/ggpubr/index.html>) packages.

**Supplementary Figure Legends**

**Supplementary** **Figure S1.** Results from the pilot study for Experiment 1. (**a**) Perceived pleasantness of the odour stimuli. (**b**) Perceived intensity of the odour stimuli. Error bars represent standard error of the mean.

**Supplementary** **Figure S2.** Odour ratings of Experiment 1 without excluding the methional odour. Perceived pleasantness of the odour stimuli. (**b**) Perceived intensity of the odour stimuli. Error bars represent standard error of the mean.

**Supplementary** **Figure S3.** Results of Experiment 1 as a function of odour. (**a**) Rates of failed-to-No-Go responses. (**b**) Rates of failed-to-Go responses. (**c**) Reaction times of correct Go responses. Error bars represent standard error of the mean.

**Supplementary** **Figure S4.** Results from the pilot study for Experiment 2. (**a**) Perceived pleasantness of the odour stimuli. (**b**) Perceived intensity of the odour stimuli. Error bars represent standard error of the mean.

**Supplementary** **Figure S5.** Odour ratings of Experiment 2 without excluding the civet odour. Perceived pleasantness of the odour stimuli. (**b**) Perceived intensity of the odour stimuli. Error bars represent standard error of the mean.

**Supplementary** **Figure S6.** Results of Experiment 2 as a function of odour. (**a**) Rates of failed-to-No-Go responses. (**b**) Rates of failed-to-Go responses. (**c**) Reaction times of correct Go responses. Error bars represent standard error of the mean.

**Supplementary Figure S7.** Rates of failed-to-No-Go responses as a function of preceding Go trials in Experiment 1. Error bars represent standard error of the mean.
